# Enhancing acute flaccid paralysis surveillance through the use of pictorial surveillance reminder cards during supplementary immunization activities, December 2014: a survey in Jigawa State, Nigeria

**DOI:** 10.11604/pamj.supp.2021.40.1.19647

**Published:** 2021-11-12

**Authors:** Lilian Akudo Okeke, Ndadilnasiya Waziri, Saheed Gidado, Joel Adegoke, Aboyowa Edukugho, Jibrin Idris, Samuel Luka Abbot, Belinda Vernyuy Uba, Adefisoye Adewole, Olufemi Ajumobi, Patrick Nguku, Oladayo Biya, Lisa Esapa, Omotayo Bolu, Eric Wiesen, Chima Ohuabunwo

**Affiliations:** 1 National Stop Transmission of Polio, African Field Epidemiology Network, Abuja, Nigeria,; 2 Nigeria Field Epidemiology and Laboratory Training Programme, African Field Epidemiology Network, Abuja, Nigeria,; 3 Centers for Disease Control and Prevention, Global Immunization Division, Atlanta, United States,; 4 African Field Epidemiology Network, Kampala, Uganda

**Keywords:** Acute flaccid paralysis, surveillance, immunization, reminder card, Nigeria, supplemental immunization activities

## Abstract

**Introduction:**

Acute flaccid paralysis (AFP) pictorial surveillance reminder cards (AFP cards) could aid AFP case identification during supplementary immunization activities (SIAs). We assessed the availability and utilization of AFP cards among vaccination teams during the December 2014 polio SIAs in Jigawa State, Nigeria.

**Methods:**

We conducted a cross-sectional survey of a convenience sample of 95 vaccination team supervisors. We used a semi-structured interviewer-administered questionnaire to collect information on socio-demographics, knowledge of AFP cases, availability and utilization of the AFP cards for case identification and investigation and non-compliance resolution by vaccination teams. Univariate and bivariate analyses were performed using Epi Info version 3.5.1.

**Results:**

Of the 95 supervisors interviewed, 86 (91%) reported that vaccinators properly displayed the AFP cards, 90 (95%) reported use of cards for AFP case identification, 88 (93%) reported use of cards to resolve non-compliance with polio vaccination and 77 (81%) reported use of cards to ask caregivers six key questions to prevent missed children. Fifty-eight (61%) supervisors knew the AFP case definition. A total of 21 possible AFP cases were identified by vaccination team members with the aid of the cards, of which 17 (81%) were referred to the nearest health facility.

**Conclusion:**

The survey demonstrated usefulness of reminder cards for identification and follow-up of AFP cases. Based on these findings, use of AFP cards was implemented in all Nigerian States and similar cards were developed and implemented for measles surveillance during SIAs.

## Introduction

Surveillance activities are critical to monitor disease trends, guide disease prevention programs and guide immediate outbreak response activities [1]. The key approach used to detect transmission of wild poliovirus (WPV) is active surveillance for acute flaccid paralysis (AFP) with laboratory testing of stool samples for the presence of poliovirus [2]. In Nigeria, the standard AFP surveillance network of surveillance officers at State and local government area (LGA) levels includes surveillance focal points at the health facility (HF) level and community informants at the settlement level. Persistent poliovirus transmission can remain undetected in populations with low population immunity if there are gaps in the surveillance system [3]. Surveillance officers are required to regularly contact HF focal points and clinicians in order to identify AFP cases. However, this active surveillance may not optimally detect all cases seen in facilities if there is poor knowledge of the AFP case definition, poor documentation of suspected AFP cases, inadequate clinician and community sensitization on AFP surveillance; or if it is not conducted due to inadequate funds for field visits, hard-to-reach populations, or the threat of violence in insecure areas [4]. These gaps can result in delayed detection of AFP cases and delayed implementation of supplementary immunization activities (SIAs) to interrupt transmission of poliovirus.

The National Stop Transmission of Polio (NSTOP) program provides technical and management support to the Polio Eradication Initiative (PEI) in Nigeria [5]. One of NSTOP´s core priorities is to strengthen AFP surveillance. Since its inception in August 2012, NSTOP has implemented activities to enhance AFP surveillance in all NSTOP-supported states [6]. These include holding community sensitization meetings on AFP case identification, conducting surveillance supervision to health facilities, and expanding AFP reporting networks by engaging community and special informants in underserved and security-compromised areas. In addition, a short message service (SMS)-based reporting system to improve AFP case reporting was implemented in Sokoto State [7]. In 2014, a polio field census of underserved populations in 209 LGAs across 17 high-risk states detected un-reported AFP cases [6].

**Figure 1:**
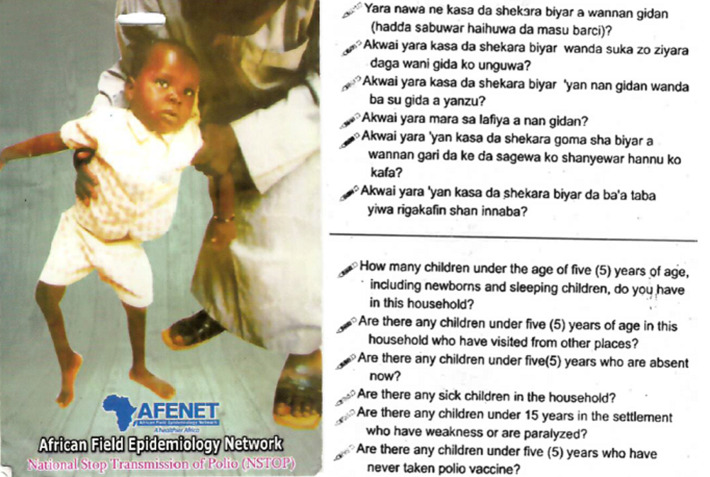
NSTOP developed and printed AFP surveillance reminder card showing an AFP case on the front page and the 6 key questions at the back

Suspected AFP cases are often identified during SIAs by vaccination teams, who travel door to door to administer oral poliovirus vaccine (OPV). To support AFP case identification, NSTOP distributed pictorial “AFP surveillance reminder cards” (Figure 1) to vaccination teams in the 184 NSTOP-supported LGAs in 12 northern states in Nigeria (Jigawa, Borno, Kaduna, Taraba, Yobe, Bauchi, Adamawa, Kano, Zamfara, Sokoto and Katsina). The AFP surveillance reminder cards are attached to a neck lanyard and have six key questions to reduce missed children during polio SIAs and identify potential AFP cases; the one relevant to AFP surveillance is: “Are there any children less than 15 years in the settlement who have weakness or are paralyzed?”

Following the distribution of these cards, NSTOP conducted a limited survey to assess the vaccination team supervisors´ knowledge of the AFP case definition and factors associated with that knowledge, and their perception of the use of the AFP card by vaccination teams.

## Methods

Jigawa State in north-central Nigeria has an estimated population of 872,200 children younger than 5 years, the target age group for OPV during SIAs. It has 27 LGAs, of which eight (Babura, Birnin Kudu, Gwaram, Kiyawa, Maigatari, Miga, Sule Tankarkar and Kazuare) have NSTOP staff embedded within the LGA immunization team.

***Study design:*** a cross-sectional survey assessed whether AFP cards help identify possible AFP cases, extending the reach of surveillance during SIAs. These cards were tested during SIAs in December 2014.

***Study population:*** the house-to-house vaccination team included a team supervisor, a vaccinator, a recorder and a community leader. Women are usually selected as the vaccinator to allow access to houses during campaigns. In accordance with the Hausa tradition, men are not allowed entry into the home of a non-relative unless the male head of household is at home and allows it. The survey population was the supervisors of vaccination teams deployed to select NSTOP-supported LGAs in Jigawa State. He/she answered questions about use of reminder cards based on observed interactions between vaccinators and caregivers.

***Sampling technique:*** a ward and settlement known to have high rates of non-compliance to polio vaccination according to SIA administrative data were selected arbitrarily within each of the 27 LGAs. A convenience sample of 2-4 team supervisors was selected in each ward, totaling 95 team supervisors.

***Methods for use of cards:*** at each house, the vaccinator or supervisor is to review the card with the caregiver and ask six questions as shown in Figure 1. Non-compliance was defined as the caregiver´s initial refusal to administer OPV to a child in the household. In cases of non-compliance, the picture on the card could be used to demonstrate the effects of polio on an unprotected child and educate the caretaker on the risks of non-vaccination.

***Data collection:*** using an interviewer-administered semi-structured questionnaire, data collected by the NSTOP LGA Officers (NSLOs) included socio-demographic information of vaccination team supervisors (age, sex, educational status and occupation), knowledge of AFP case definition, suspected AFP reporting process by vaccination teams, availability and the utilization of the AFP cards among the vaccination teams and perceptions of the utility of the AFP cards.

***Data processing and analysis:*** data were entered and analyzed using Epi Info version 3.5.1 and Microsoft Excel to determine frequencies and proportions. Bivariate analyses were performed and Chi square test was used to determine significant association, with statistical significance placed at p value < 0.05.

***Ethical consideration:*** ethical clearance was obtained from the Jigawa State Primary Health Care Development Agency (SPHCDA) board. Final approval to conduct this survey was obtained from the National Coordinator, NSTOP Program, African Field Epidemiology Network, Nigeria Country Office.

## Results

Ninety-five team supervisors were enrolled in the survey. All team supervisors responded (100%). The mean age of respondents was 24 years (standard deviation 4.6 years). Ninety-three respondents were females (98%). Seventy-one (75%) had secondary level of education, and 25 (26%) were unemployed when there are no polio SIAs (Table 1).

**Table 1:** socio-demographics characteristics of vaccination team supervisors in NSTOP- supported LGAs, during December Supplementary Immunization Activity, Jigawa State, Nigeria, 2014

Characteristics	Frequency n=95	Percent
**Age group(years)**		
≤30	88	92.6
>30	7	7.0
Mean age 24 ± 4.6		4
**Sex**		
Female	93	97.9
Male	2	2.1
**Educational status**		
Secondary	71	74.7
Tertiary	24	25.3
Occupational status	4	4.2
Civil Servant	21	22.1
Homemaker Students	41	43.2
Self employed	4	4.2
Unemployed (when there are no polio SIAs)	25	26.3

Abbreviations: NSTOP = National Stop Transmission of Polio, LGAs = Local Government Area, SIA = Supplementary Immunization Activity

Of the 95 respondents, 86(91%) observed vaccinators wearing the pictorial AFP surveillance reminder cards around their neck, 77(81%) observed vaccinators using the cards to ask mothers and caregivers the six key questions, 90(95%) indicated that teams used the cards for identifying potential AFP cases and 88(93%) used the cards to resolve issues of non-compliance with polio vaccination (Table 2). Fifty-eight (61%) supervisors knew the AFP case definition, 86(90.5%) knew the importance of enquiring for an AFP case, 24(25%) knew to whom to report an AFP case and 84 (88.4%) knew the reason for use of the cards. In addition, 92(97%) believed the card was useful for AFP identification and 88 (93%) believed the cards aided in resolving issues of non-compliance (Table 3). A total of 21 possible AFP cases were identified using the vaccination team supervisors during the December 2014 SIAs; 17(81%) of those were referred for investigation.

**Table 2:** supervisor-reported availability and utilization of AFP surveillance reminder cards among vaccinators during December Supplementary Immunization Activity, Jigawa State, Nigeria, 2014

Observations	Frequency n=95	Percent
Persons seen properly displaying AFP surveillance reminder cards (i.e. hung on an lanyard around the neck)	86	90.5
Used cards for AFP identification	90	94.7
Used cards to resolve non-compliance with polio vaccination among care givers	88	92.6
Used cards to ask the 6 key questions	77	81.1

Abbreviations: AFP = Acute Flaccid Paralysis, SIA = Supplementary Immunization Activity

**Table 3:** knowledge and perception among Supplementary Immunization Activities team supervisors regarding AFP reminder cards during December SIAs, Jigawa State, Nigeria, 2014

Characteristics	Frequency n=95	Percent
**Knowledge on AFP and pictorial AFP surveillance reminder cards**		
Knew AFP case definition	58	61.1
Knew the importance of enquiring for AFP cases	86	90.5
Knew the reasons for use of the AFP reminder cards	84	88.4
Knew to whom to report an AFP case	24	25.0
**Perception on the use of the pictorial AFP surveillance reminder cards**		
Believed AFP reminder card is useful for AFP identification	92	96.8
Believed AFP reminder card aided in resolving non-compliance	88	92.6

Abbreviations: AFP= Acute Flaccid Paralysis, SIA = Supplementary Immunization Activity

In bivariate analysis, the knowledge level of the supervisors about the AFP case definition was found not to be associated with age, sex, educational status and occupation of respondents. Their perception of the AFP surveillance reminder cards aiding in resolving non-compliance was found to be associated with their teams´ observed utilization of the cards during SIAs (p < 0.05) (Table 4).

**Table 4:** association between knowledge on AFP cases definition, perception of vaccination team supervisors on the use of AFP reminder cards and observed utilization of AFP cards during December SIAs, Jigawa state, Northern Nigeria, 2014 n=95

Characteristics	Observed use of AFP reminder cards		X^2^	p value
	Yes n (%)	No n (%)		
**Knowledge on AFP and AFP reminder cards**				
**Knew AFP case definition**			0.15	0.70
Yes	54 (60.9)	36 (39.1)		
No	4 (80.0)	1 (20.0)		
**Knew the importance of enquiring for suspected AFP cases**				
Yes	82 (91.1)	8 (8.9)	0.0017	0.97
No	4 (80.0)	1 (20.0)		
**Knew the importance of the AFP reminder cards**				
Yes	81 (90.0)	9 (10.0)	1.75	0.19
No	3 (60.0)	2 (40.0)		
**Perception on the use of the AFP cards**				
**Believed pictorial AFP reminder card is important**				
Yes	88 (97.8)	2 (2.2)	0.81	0.37
No	4 (80.0)	1 (20.0)		
**Believed pictorial AFP reminder card aided in resolving non-compliance**				
Yes	85 (94.4)	5 (5.6)	3.96	0.04
No	3 (60.0)	2 (40.0)		

Abbreviations: AFP= Acute Flaccid Paralysis, SIA = Supplementary Immunization Activity

## Discussion

Vaccinators can use a simple tool to conduct AFP surveillance during SIAs, thereby extending the reach of the surveillance system. During trainings for SIAs, vaccinators are instructed to ask about any recent events of paralysis to identify potential AFP cases that were not previously identified through the standard AFP reporting network. To our knowledge, the survey is the first to assess usefulness of reminder cards by house-to-house vaccination teams to strengthen AFP surveillance and improve uptake of OPV in initially non-compliant households. The survey revealed a gap in the team supervisors´ knowledge of the standard AFP case definition, which is key for AFP identification, even though the pictorial on the AFP reminder card may have aided in AFP identification. Although this survey found that pictorial AFP surveillance reminder cards could be useful for identification of possible AFP cases during SIAs, four possible AFP cases identified by the teams were not referred for investigation. This could have been due to limited knowledge to whom cases should be reported. This knowledge gap should be addressed by training vaccination teams on the need and means for referral of all AFP cases identified during the ward level training in preparation for the OPV campaign.

Our findings suggest that vaccinators utilizing the AFP card photo helps caregivers to see what AFP looks like and to appreciate the danger of non-vaccination. Our survey was conducted in settlements known to have high rates of non-compliance to polio vaccination. Supervisors reported unquantified non-compliance among caregivers during the December 2014 polio SIAs; supervisors´ responses indicated that an unquantified number of instances were resolved when teams showed the picture on the reminder cards to the caregivers and reinforcing the risks of non-vaccination.

Many studies have demonstrated that use of simple tools could improve reporting of AFP cases, other diseases and health interventions [7-9]. Datta et al., demonstrated that the use of Short Message Services (SMS) reminders could improve AFP reporting, and recommended that SMS could be adapted to improve AFP surveillance and reporting post-eradication in high-risk countries for poliovirus importation or emergence and circulation of vaccine-derived polioviruses [9]. Similarly, Gurol et al. demonstrated that mobile phone messaging applications, such as SMS and Multimedia Message Service (MMS), could provide an important, inexpensive delivery medium for reminders for healthcare appointments [10]. A systematic literature review study, conducted by Rathbone and Prescoth, 2017 demonstrated that the use of simple tools like mobile apps and SMS text messaging for physical and mental health interventions are promising and efficient [11]. During the smallpox eradication initiative, pictorial cards were used to aid surveillance.

This survey had many limitations. First, this survey was limited to one State and used a convenience sample of LGAs, wards, settlements and respondents. Second, the supervisors could have exaggerated the positive use of reminder cards for AFP case identification without actual observation. Third, there were inconsistencies in some of the responses (e.g., more supervisors indicated that they believed the reminder cards were useful for AFP identification than knew the reasons for use of the reminder cards). Fourth, we have no quantitative data on how many non-compliant households were persuaded to accept vaccination based on the use of reminder cards and therefore its value for this purpose. Lastly, some of the potential AFP cases identified may have been previously reported and investigated.

## Conclusion

Our survey findings support the positive use of the AFP reminder cards during the December 2014 SIA. Based on identification of 17 potential AFP cases referred for investigation, there may be value in using the cards for extending the reach of the standard AFP surveillance system. Based on our findings, the use of AFP reminder card during SIAs was extended to all States in Nigeria. The card has been adapted for measles surveillance as well.

### What is known about this topic

- Acute Flaccid Paralysis surveillance is one of the key strategies for polio eradication. There are limitations in AFP surveillance in areas of Nigeria.

### What this study adds

- Pictorial AFP reminder card used during OPV vaccination campaigns could help reduce non-compliance to vaccination and potentially enhance surveillance of AFP.
